# Establishment of a Community Advisory Board in Lima, based on the adaptation of the Patient Advisor Toolkit to enhance recruitment in dementia research

**DOI:** 10.1002/alz70860_105831

**Published:** 2025-12-23

**Authors:** Belen Custodio, Rosa Montesinos, José Carlos Huilca, Diego Bustamante‐Paytan, Katherine Agüero‐Flores, Graciet Verastegui, Marco Malaga, Nilton Custodio

**Affiliations:** ^1^ Unidad de Investigación de Deterioro Cognitivo y Prevención de Demencia, Instituto Peruano de Neurociencias, Lima, Lima, Peru; ^2^ Unidad de Investigación y Docencia, Equilibria, Lima, Peru., Lima, Lima, Peru; ^3^ Unidad de Investigación de Deterioro Cognitivo y Prevención de Demencia, Instituto Peruano de Neurociencias, Lima, Peru, Lima, Lima, Peru

## Abstract

**Background:**

Community Advisory Boards (CABs) are formal groups of community members who collaborate with research teams. The establishment of CABs have been shown to be an effective strategy for enhancing community engagement in research. This study aims to illustrate the process of establishing a Community Advisory Board in Lima, Peru, based on the adaptation of the *Patient Advisor Toolkit*, to involve the community in designing dissemination and recruitment strategies for dementia research studies.

**Method:**

This retrospective study describes the creation of a CAB in Lima‐Peru, through the adaptation and translation of the *Patient Advisor Toolkit* developed by the Wisconsin Network for Research Support. This tool provides a step‐by‐step guide for researchers facilitating the sessions, enabling community members and research teams to collaborate effectively. Prior to the board's first meeting, facilitators received training, which included reviewing materials and engaging in role‐play exercises.

**Result:**

The CAB is composed of four women and two men from diverse backgrounds. The group includes two caregivers of dementia patients, a pharmaceutical sales representative, and three individuals with no prior connection to dementia research studies or a medical background. According to the *Patient Advisor Toolkit*, training for CAB members should occur during the first meeting. However, in this case, the training was divided into two sessions held one week apart to ensure the information provided to the board members was more manageable. During these initial meetings, it was discussed the definition and functions of a CAB, board's members established their rules and responsibilities, outlined their expectations of the research team, agreed to meet every three months, and participated in a brief simulation of regular meetings. This simulation allowed them to provide constructive feedback on materials and ideas presented by the research team.

**Conclusion:**

By adapting and translating the *Patient Advisor Toolkit*, this study provided a structured approach to CAB formation, ensuring effective collaboration between community members and researchers. This model may serve as a reference for integrating community perspectives into dementia research in similar settings.

